# PpINH1, an invertase inhibitor, interacts with vacuolar invertase PpVIN2 in regulating the chilling tolerance of peach fruit

**DOI:** 10.1038/s41438-020-00389-8

**Published:** 2020-10-01

**Authors:** Xingxing Wang, Yi Chen, Shu Jiang, Feng Xu, Hongfei Wang, Yingying Wei, Xingfeng Shao

**Affiliations:** College of Food and Pharmaceutical Sciences, Ningbo University, 315800 Ningbo, China

**Keywords:** Abiotic, Population genetics, Abiotic, Population genetics

## Abstract

Sucrose metabolism, particularly the decomposition of sucrose by invertase, plays a central role in plant responses to cold stress. Invertase inhibitors (INHs) evolved in higher plants as essential regulators of sucrose metabolism. By limiting invertase activity, INHs keep cellular sugar levels elevated, which provides enhanced protection to plants under stress. Our results showed that the expression of *PpVIN2*, the only vacuolar invertase (VIN) gene in peach fruit sensitive to chilling temperatures, increases significantly during cold storage, while VIN enzyme activity increases more modestly. We also found that peach fruit transiently overexpressing *PpINH1* had decreased VIN activity. Interactions of PpINH1 and PpVIN2 with recombinant proteins were shown by yeast two-hybrid assays and bimolecular fluorescence complementation assays, as well as in vitro. During cold storage, trehalose-treated peach fruit had significantly increased *PpINH1* expression, decreased VIN enzyme activity, and significantly higher sucrose content than did untreated fruit. As a result, the treated fruit had enhanced resistance to chilling injury. Collectively, our data show that the post-translational repression of VIN enzyme activity by PpINH1 helps maintain sucrose levels in peach fruit during cold storage, thereby improving resistance to chilling injury.

## Introduction

Damage from low-temperature stress is responsible for significant economic losses in commercial agriculture. Sugars play a predominant role in protecting plants from damage due to low temperatures because they regulate osmotic pressure, stabilize membrane structures, eliminate reactive oxygen species, and function as signaling molecules^1^. Peach (*Prunus persica* L. Batsch) fruit, like many fruits that are sensitive to low temperatures, develop chilling injuries after storage for 1 or 2 weeks at 2–5 °C^2^. The content of sucrose, the major sugar in peach fruit, decreases after harvest, while the reducing sugar content increases^3^. Sucrose decomposition and the continued demand for sugar in peach fruit at low temperatures affect their sensitivity to cold^4^. Sucrose is considered an osmotic substance in regulating the plant response to different stresses^5^. Sucrose, fructose, and glucose have been reported to be signaling molecules involved in fruit development and ripening. Most importantly, emerging evidence has shown that cold tolerance in plants is related to sucrose metabolism, as respiration increases in response to sucrose decomposition products, glucose and fructose^6^. In peach, maintaining higher sucrose concentrations may balance energy savings and respiration to improve cold resistance. Previously, we found that sucrose degradation increases in peach fruit subjected to chilling stress and that maintenance of high-sucrose levels improves membrane stability and resistance to cold stress^7,8^.

Invertases are classified according to their pH optima as acidic, alkaline, or neutral invertases. Acid invertase (AI) is thought to be the most important enzyme in fruit sucrose metabolism because it controls the composition of sugars and affects the response to stress^9,10^. AI is further subdivided (in terms of its subcellular localization) into cell wall-bound invertase (CWIN) and vacuolar acid invertase (VIN); the latter is also known as soluble AI. Multiple invertase genes are found in most plants, and peach contains 2 VIN and 5 CWIN genes^11^. Evolutionary analyses have shown that CWINs exhibit more sequence variability than do VINs, suggesting that they share a common VIN ancestor^12^.

VINs catalyze the irreversible decomposition of sucrose into fructose and glucose, thereby helping to establish and maintain cell osmotic potential and protect plants from environmental stresses^12^. Generally, VINs are significantly upregulated in plants under cold stress, but VIN activity does not increase proportionately with gene expression^13^. In peach, only *PpVIN2* expression is sensitive to low temperature^11^. Although *PpVIN2* expression increases substantially at cold temperatures, VIN activity increases to a much lower degree—approximately 2-fold^11^. This result might suggest that VIN activity is regulated by a post-transcriptional mechanism. To our knowledge, there are different mechanisms involved in the regulation of invertase expression or activity, including transcriptional regulation^14^, exon skipping^15^, protein inhibition^16^, and post-transcriptional modification^17^.

Invertase inhibitors (INHs) were first identified in the 1960s as endogenous proteins that inhibit invertase activity in potato (*Solanum tuberosum*)^18^. They have since been shown to interact with VINs and regulate their activity, thereby playing a vital role in sugar signaling and carbon allocation^12^. INHs are members of the pectin methylesterase inhibitor-related protein family and are classified as cell wall inhibitors (CIFs) or vacuolar inhibitors (VIFs) on the basis of their subcellular location^16^. Unlike in model plant species, little is known about INHs and their physiological significance in peach fruit, and the post-translational regulation of VIN by inhibitors been not been examined in peach fruit.

To investigate whether peach INH (PpINH) functions as a post-translational regulator of VIN activity in fruit, five *PpINH* genes were cloned, and their interactions with PpVIN2 were studied using a yeast two-hybrid (Y2H) system. Based on the results of Y2H assays, the interaction between PpINH1 and PpVIN2 was further determined via biomolecular fluorescence complementation (BiFC) assays in tobacco. PpINH1 function was also investigated in peach fruit using an *Agrobacterium*-based transient expression system. Finally, PpINH1 and PpVIN2 proteins were prepared using heterologous expression systems, and interactions between the proteins were studied in vitro.

Trehalose (α-d-glucopyranosyl-1,1-αD-glucopyranoside) is a nonreducing glucose disaccharide that is synthesized under abiotic stress during plant development^19,20^. Such synthesis can produce trehalose-6-phosphate, an intermediate compound that has been widely shown to serve as an effective sucrose sensor, having direct effects on the type of response to various environmental conditions^21^. Exogenous trehalose treatment has been used to enhance the cold tolerance of crops, harvested fruits^21^, and fresh-cut peppers^22^. Here, we treated peach fruit with exogenous trehalose to investigate its effect on chilling injury (CI), sucrose content, VIN activity, *PpVIN2* expression, and *INH* gene expression. The results demonstrated that the application of exogenous trehalose decreased sucrose metabolism in and CI of peach fruit subjected to cold stress.

## Results

### Analysis of INH protein evolution

**Fig. 1:**
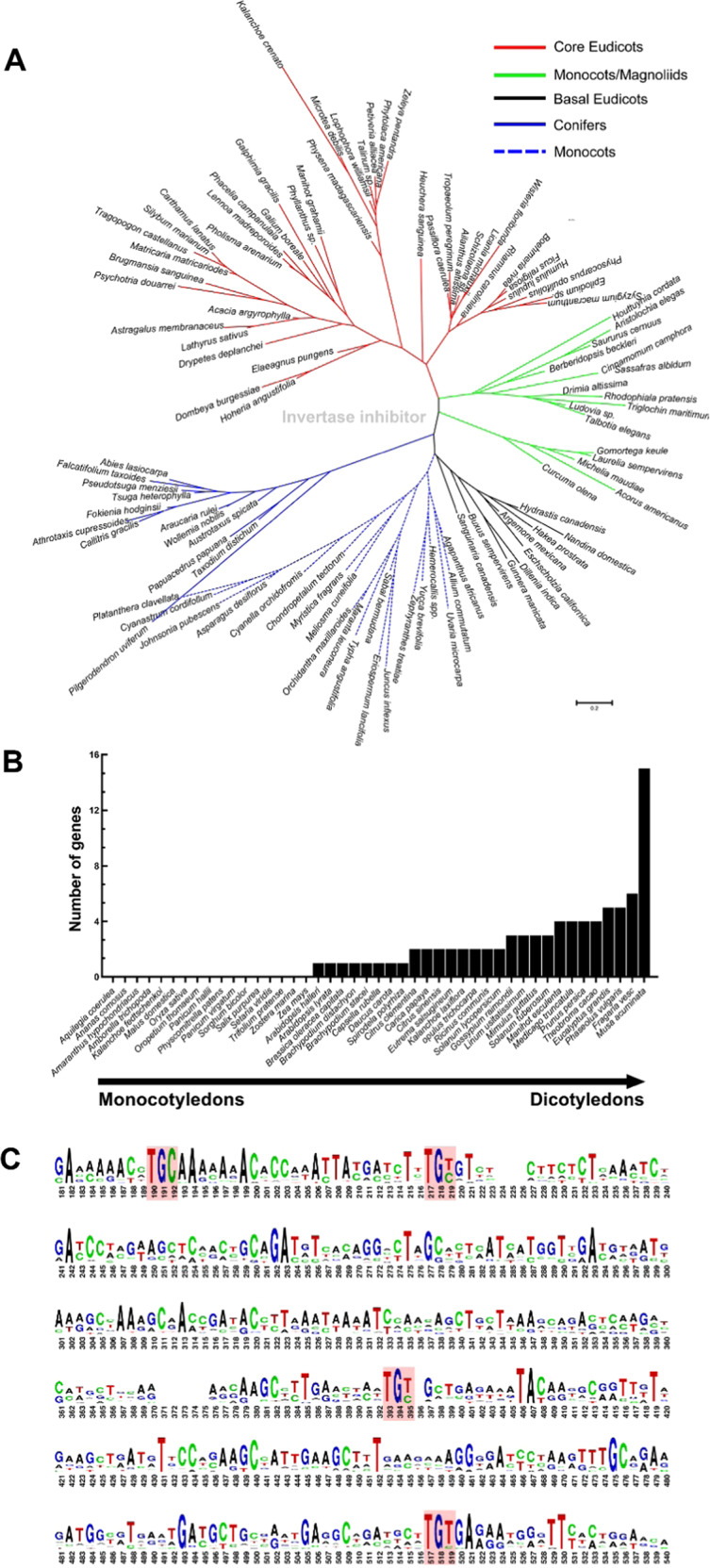
INH evolution in green plants. **a** Phylogenetic tree constructed using INH proteins from 100 representative green plant species. **b** INH gene copy number in 45 flowering plant species. **c** Conservation of INH genes in 45 plants. Codons that encode the four highly conserved cysteine residues are shown in boxes, and the height of the logo indicates the residue frequency

To analyze the evolution of INH in green plants, the PpINH1 protein sequence was used to conduct a BLASTP search for similar proteins whose sequence is deposited in the One Thousand Plant Transcriptomes database (*E*-value < 10^−10^). More than 2000 target sequences were identified. To construct a detailed evolutionary tree, 100 representative plant species were chosen randomly, and INH evolutionary analysis was then conducted (Fig. 1a). INH was found only in flowering plant species, including conifers, monocots, and eudicots. To analyze the evolution of INH in more detail among angiosperms, we used BLASTP to query the Phytozome 12.1 database to define a smaller number of representative flowering plant species, after which we counted the number of INH genes in each one (Fig. 1b). It is interesting to note that no INHs were detected in *Amborella trichopoda*, which is the common ancestor of living angiosperms. Variations in the number of INH genes were also observed, presumably reflecting gene duplication and loss events that have occurred during speciation, and there was a clear difference between dicots and monocots (Fig. 1b). Analysis of the *INH* genes at the nucleic acid level showed that the first of 4 conserved cysteine sites are encoded as “TGC” in all species. However, the other conserved cysteine codons are more heterogeneous (Fig. 1c).

### Promoter analysis of *PpINH*s

**Fig. 2:**
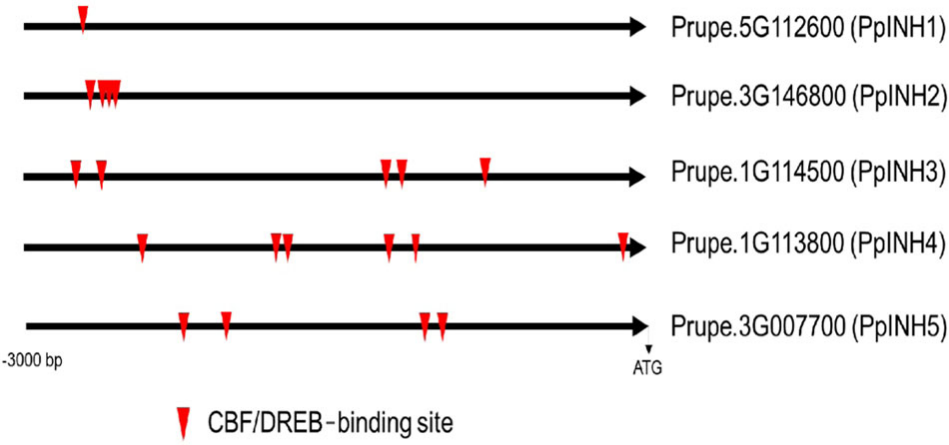
Promoter analysis of PpINHs. Promoter regions are 3000 bp in length directly upstream of the first codon. Putative CBF/DREB (C-repeat binding factor/dehydration-responsive-element)-binding sites are indicated by arrowheads

VIN regulates sucrose metabolism in conjunction with INH-interacting proteins to enable plants to resist cold stress. To determine whether *PpINH* gene expression is sensitive to low temperature, *PpINH* promoters (defined as regions 3000 bp in length directly upstream from *PpINH* coding sequences) were examined in the peach genome. CBF/DREB (C-repeat binding factor/dehydration-responsive-element)-binding sites are present within each *PpINH* promoter (Fig. 2). The CBF/DREB protein binds specifically to G(TG)CGG cis-elements involved in regulating plant responses to drought, low temperature, and stress. These results support the hypothesis that INH proteins act as key regulators in flowering plants to mediate the response to cold stress, especially in dicots.

### PpINH1 interacts with PpVIN2 in the Y2H system

**Fig. 3:**
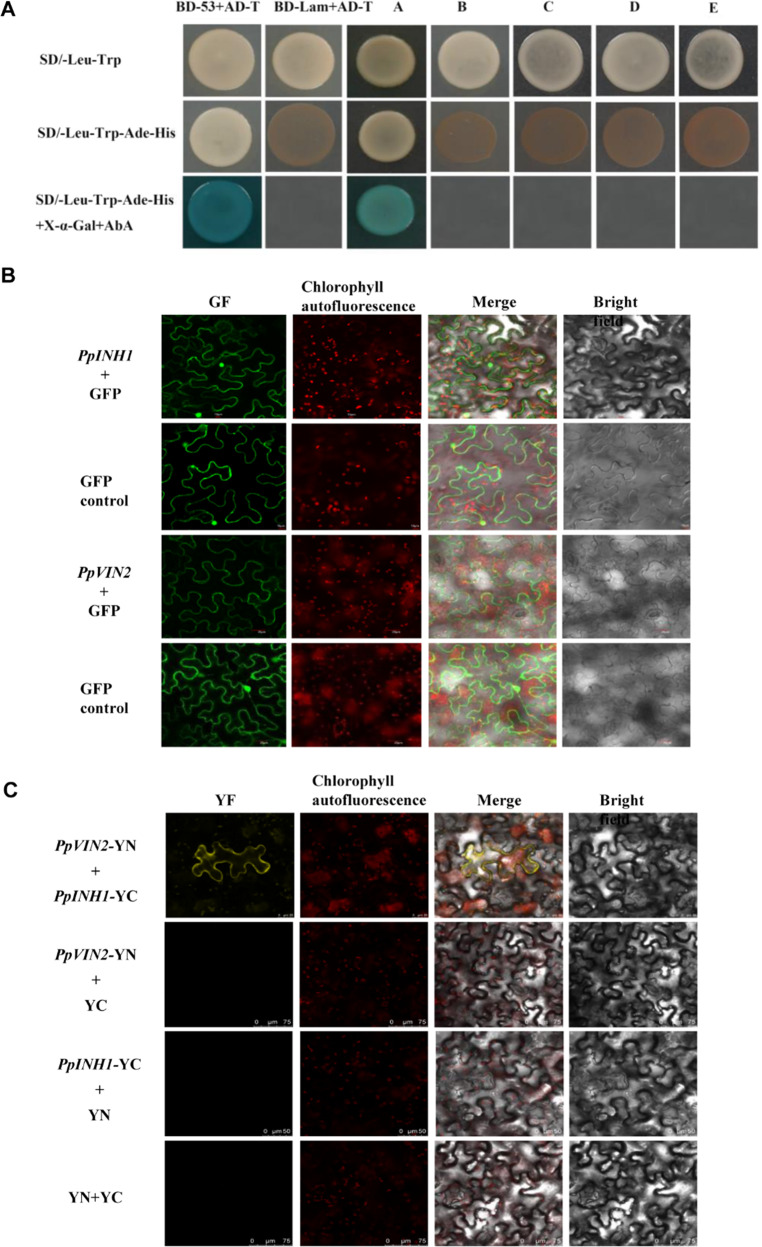
Interaction analysis of PpVIN2 and PpINH1-5 in vivo. **a** Interaction of five PpINH proteins and PpVIN2 analyzed using a Y2H system. The yeast strain was cotransformed with PpVIN2-pGBKT7 (BD) and PpINHx-pGADT7 (AD). BD-p53 and AD-T were used as positive controls, while BD-Lam and AD-T were used as negative controls. Yeast cells were spotted onto solid media (composition shown on left) and then incubated at 30 °C for 72 h. Columns A–E correspond to cells expressing PpINH1, 2, 3, 4, and 5, respectively. Interaction was detected only in column A (PpINH1). **b** Subcellular localization was visualized for both proteins in the tobacco leaves. The empty vector served as a negative control. The scale bars represent 10 μm to 20 μm. **c** BiFC analysis of the interaction between PpINH1 and PpVIN2 in tobacco epidermal cells. Combinations of *PpVIN2*-YN with YC, *PpINH1*-YC with YN, and YN with YC served as negative controls. YC, C-termini of YFP; YN, N-termini of YFP. The scale bars represent 25 μm to 75 μm

When PpVIN2 was used as “bait”, each inhibitor protein (“prey”) was tested for its ability to interact with PpVIN2 in a Y2H system. Briefly, a positive result occurs when the bait and prey proteins interact to bring a DNA-binding domain (BD) and an activating domain (AD) into proximity, thereby activating the transcription of reporter genes. This enables the yeast host to grow on plates that lack four specific amino acids, exhibit strong resistance to AbA, and generate blue colonies. Only PpINH1 interacted with PpVIN2 (Fig. 3a). In control experiments (data not shown), yeast cells containing either BD-*PpVIN2* or AD-*PpINH1* exhibited no self-activation or cell toxicity.

### BiFC assays show colocalization of *PpINH1* and *PpVIN2* in tobacco cells

To test PpINH1 and PpVIN2 subcellular localization, a PpINH1-GFP construct and an empty GFP vector were transformed into 1-month-old tobacco leaves. As shown in Fig. 3b, there was no difference in the localization or fluorescence distribution signal between the control GFP and PpINH1-GFP, indicating that PpINH1 was localized in the cell membrane, cytoplasm and nucleus. The fluorescent signal of PpVIN2 was observed in the membrane and cytoplasm, suggesting that PpVIN2 was located in the cell membrane and cytoplasm (Fig. 3b). When the genes were coexpressed in tobacco cells in BiFC assays, PpINH1 and PpVIN2 colocalized (Fig. 3c). No appreciable fluorescence was observed when *PpINH1*-YC was coexpressed with YN, when *PpVIN2*-YN was coexpressed with YC, or when YC was coexpressed with YN. These results support the hypothesis that PpINH1 interacts with PpVIN2 in vivo.

### Transient overexpression of *PpINH1* in peach fruit inhibits VIN activity

**Fig. 4:**
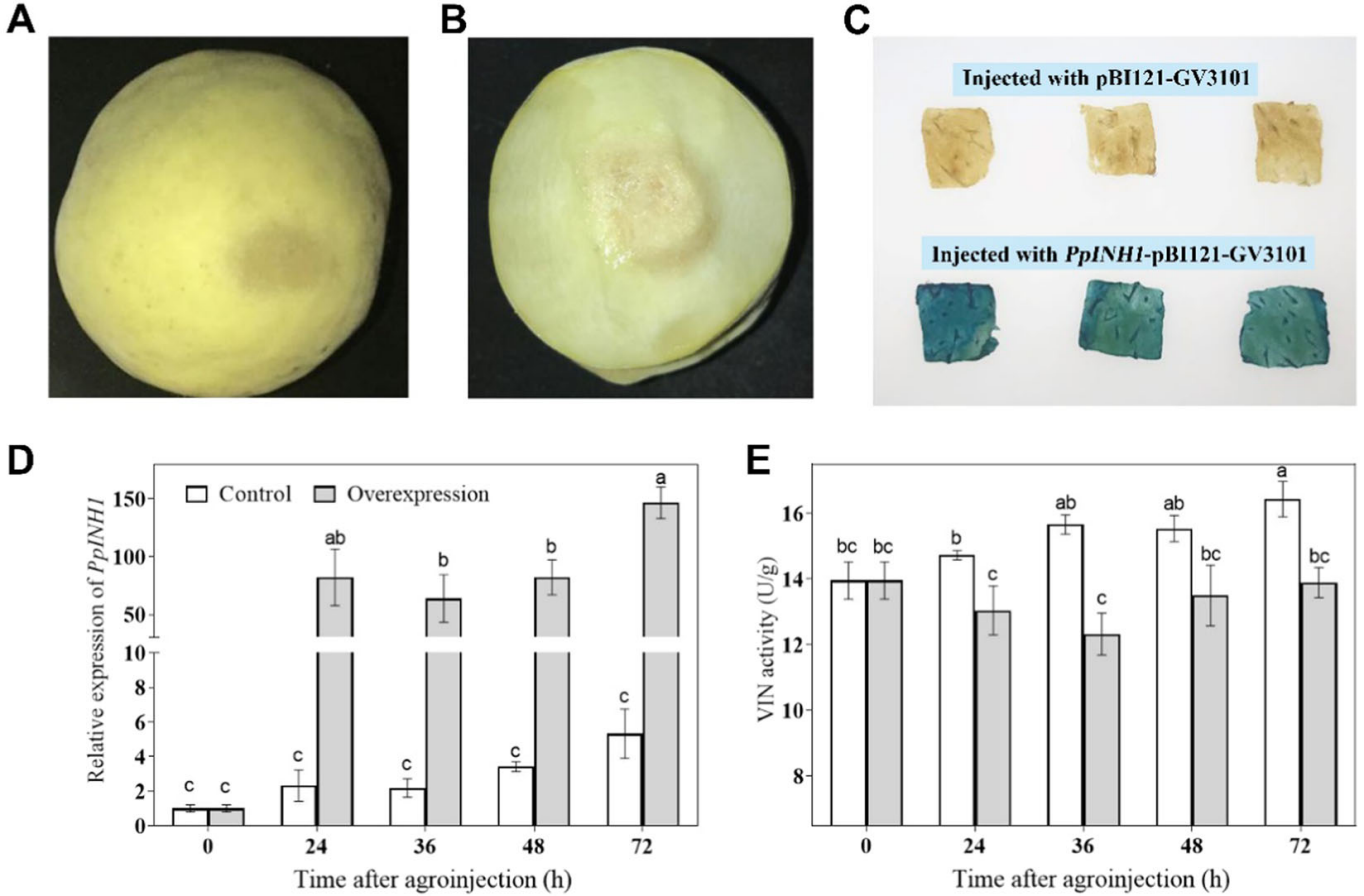
Overexpression of *PpINH1* inhibits VIN activity in vivo. Peach fruits in the green ripening stage are shown at 0 h (**a**) and 36 h (**b**) after injection with *Agrobacterium* to obtain transient expression of *PpINH1*. **c** Histochemical GUS staining of peach tissue from fruits injected with pBI121-*PpINH1*-GV3101 or pBI121-GV3101. The images were obtained 36 h after injection. The samples were imaged separately, and the images were combined to create a collage. **d** and **e** Effects of transiently overexpressed PpINH1 on the expression of *PpINH1* (**d**) and VIN activity (**e**) in peach at 0, 24, 36, 48, and 72 h after injection with *Agrobacterium*. The values are the means ± SEs, *n* = 3; the lowercase letters show significant differences at the 0.05 level according to Tukey’s LSD analysis

A pBI121-*PpINH1* overexpression construct was introduced into peach fruit via *Agrobacterium tumefaciens*-mediated injection (Fig. 4a, b). As shown in Fig. 4c, GUS staining shows that pBI121-*PpINH1* was successfully transferred into peach fruit and overexpressed. Compared with that of the control, the expression of *PpINH1* increased 47-fold, 29-fold, 11-fold, and 27-fold at 24, 36, 48, and 72 h after injection, respectively (Fig. 4d).

The VIN activity in peach fruit overexpressing *PpINH1* decreased during the first 36 h after injection and then recovered nearly to the initial levels during the remaining 48 h, although VIN activity remained significantly below that of the controls at each time point. The VIN activity in control peach fruit (injected with *Agrobacterium tumefaciens* containing the empty pBI121 vector) increased throughout the 72 h experiment (Fig. 4e).

### Expression of PpINH1 and PpVIN2 recombinant proteins in vitro

**Fig. 5:**
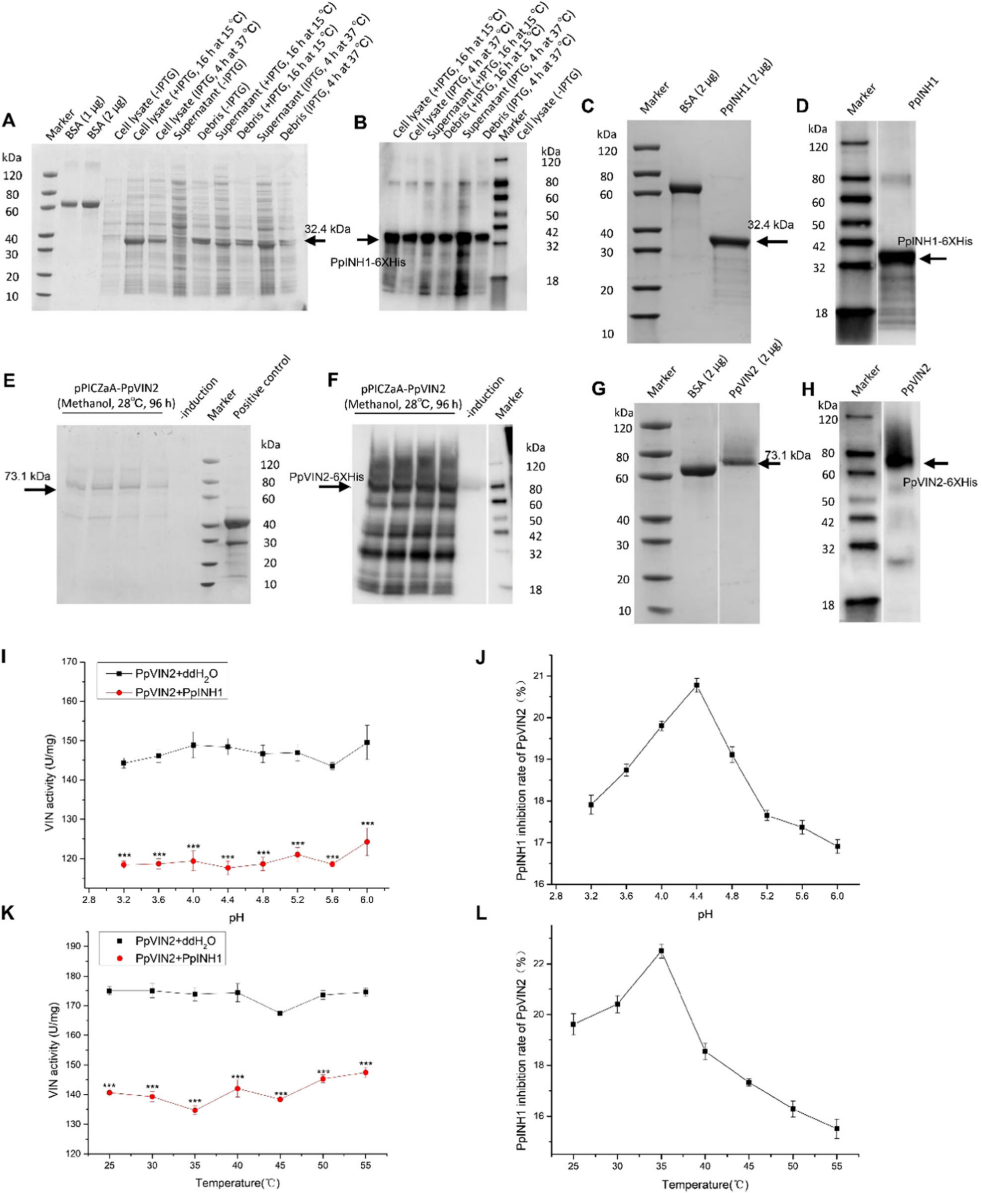
Expression, identification, and purification of PpINH1 and PpVIN2 proteins and their interaction in vitro. SDS-PAGE (**a**) and western blot analysis (**b**) of PpINH1 detected in BL21(DE3) cell fractions. SDS-PAGE (**c**) and western blot analysis (**d**) of purified PpINH1. SDS-PAGE (**e**) and western blot analysis (**f**) of PpVIN2 expressed in X-33 cells. SDS-PAGE (**g**) and western blot analysis (**h**) of purified PpVIN2. The arrows indicate the position of the recombinant protein. **i** Effect of PpINH1 on PpVIN2 activity at different pH levels. **j** Effect of PpINH1 on PpVIN2 at different pH levels, expressed in terms of inhibition rate (%). **k** Effect of PpINH1 on PpVIN2 activity at different temperatures. **l** Effect of PpINH1 on PpVIN2 at different temperatures, expressed in terms of inhibition rate (%)

The 438 bp *PpINH1* gene encodes a protein of 145 amino acids, with a theoretical molecular mass of 15.06 kDa and a predicted isoelectric point of 4.34. PpINH1 was not predicted to comprise a signal peptide (Supplementary Fig. S1a) or contain a transmembrane region (Supplementary Fig. S2a). *PpINH1* cDNA was cloned into pET-32a and expressed as an N-terminal-His-tagged protein in *Escherichia coli* (*E. coli*) BL21 (DE3). After the induction conditions were optimized (data not shown), expression was induced at 15 °C for 16 h, and the protein was recovered in the supernatant (Fig. 5a, b). The yield was ~17.7 mg/L of culture.

The *PpVIN2* CDS is 2061 nucleotides in length and encodes a protein of 686 amino acids, with a predicted molecular mass of 76.49 kDa and an isoelectric point of 6.45. PpVIN2 is not predicted to contain a signal peptide (Supplementary Fig. S1b), and the C-terminus contains a potential transmembrane region (Supplementary Fig. S2b). The molecular weight of the protein after removal of the transmembrane domain is 64 kDa. The addition of the His-tag resulted in a 73.1 kDa fusion protein (Fig. 5e, f). To conduct biochemical and molecular studies, *PpVIN2* was expressed in *Pichia pastoris* using a pPICZαA vector. After expression was induced with methanol, the recombinant protein was purified from the supernatant, with a yield of ~2.77 mg/L of culture. Samples of purified PpINH1-5×His (32.4 kDa) and PpVIN2-5×His (73.1 kDa) in polyacrylamide gels are displayed in Fig. 5c, d, g, h, respectively.

### PpINH1 inhibition of PpVIN2 is affected by pH and temperature

As shown in Fig. 5i, PpINH1 inhibition of PpVIN2 occurs across a pH range from 3.2 to 6.0, with maximum inhibition at pH 4.4 (Fig. 5j), which is close to the optimum pH for VIN activity (pH 4.5). Inhibition gradually weakens at pH values in excess of 5.2.

PpINH1 inhibition of PpVIN2 activity was also measured at temperatures ranging from 25 °C to 55 °C (Fig. 5k). As shown in Fig. 5l, inhibition was greatest at 35 °C (Fig. 5l) but decreased rapidly as temperatures increased. These results demonstrate that PpINH1 is a potent inhibitor of PpVIN2 activity and that inhibition varies with pH and temperature in vitro.

### Postharvest trehalose treatment upregulates *PpINH1* and increases the chilling resistance of peach fruit

**Fig. 6:**
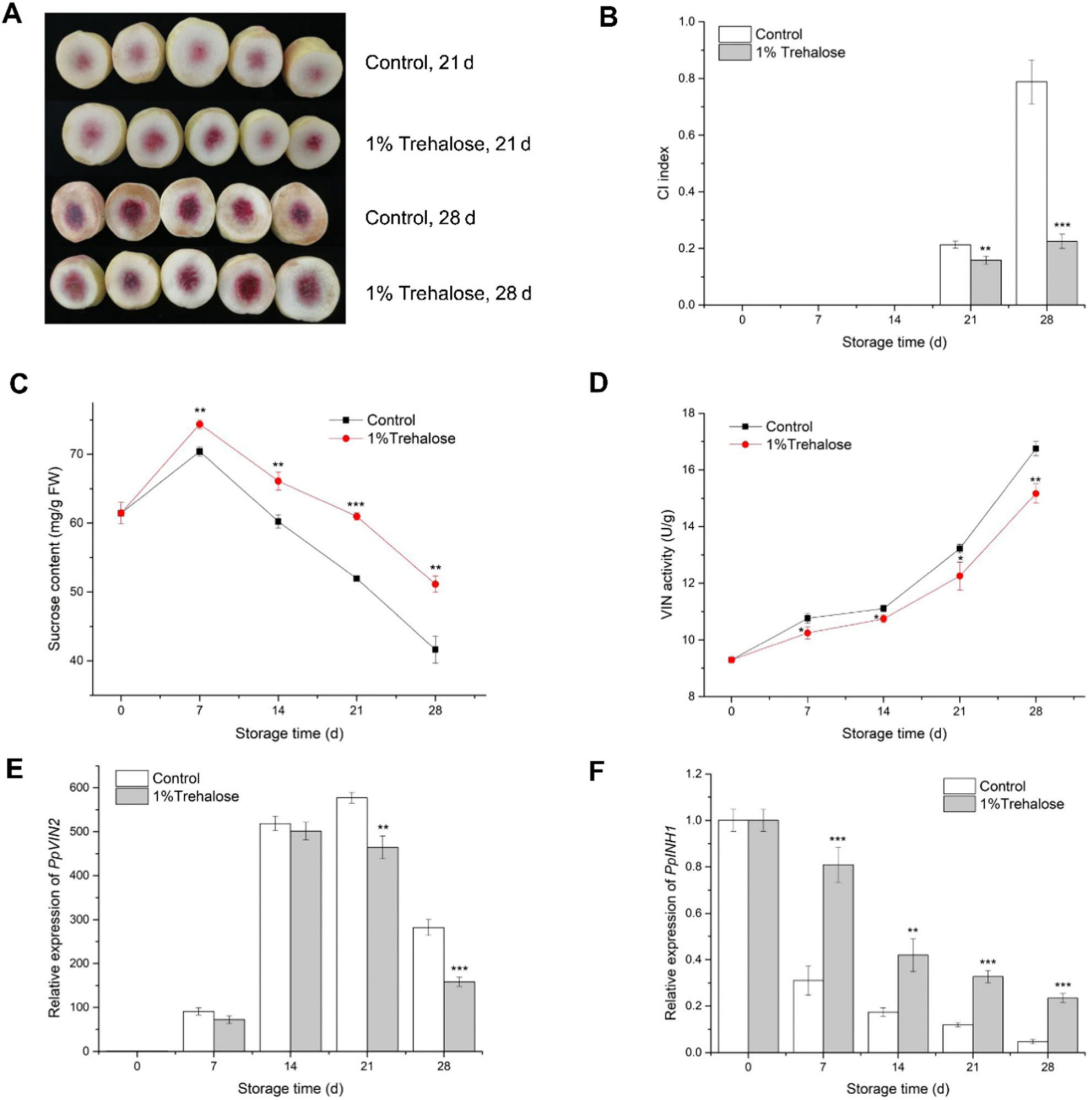
Effects of trehalose treatment on postharvest peach fruits. **a** Representative images of CI symptoms of treated and untreated peach fruit after storage for 21 and 28 days at 5 °C. **b** CI index, **c** sucrose content, **d** VIN activity, **e** *PpVIN2* expression, and **f** *PpINH1* expression in trehalose-treated and untreated peach fruit during cold storage. The values are the means ± SEs. Student’s *t*-test was used to assess differences between the treatment and control groups. “*”, “*”, “**”, and “***” indicate significant differences at *p* < 0.05, 0.01, and 0.001, respectively

CI of peach fruit occurred after 2 weeks of storage at 5 °C. Visible flesh browning was observed on day 21 of storage, and at day 28, severe CI was observed. In contrast, peach fruit treated with trehalose did not exhibit substantial CI symptoms after 21 or 28 days of storage (Fig. 6a, b). The sucrose content in trehalose-treated fruit was significantly higher than that in untreated control fruit throughout the 28-day storage period (Fig. 6c). In the control fruit, VIN activity increased nearly 2-fold during 28 days of cold storage (Fig. 6d), while *PpVIN2* expression increased by more than 500-fold by the 21st day of storage (Fig. 6e). In the trehalose-treated fruit, VIN activity also increased during storage but remained lower than that in the control fruit. *PpVIN2* expression also increased with storage time but was significantly lower than that in the control fruit on days 21 and 28. Expression of *PpINH1* in control fruit decreased significantly during storage, as did expression in trehalose-treated fruit, but *PpINH1* expression in treated peach fruit remained significantly high throughout cold storage (Fig. 6f).

## Discussion

### PpINHs are cold-responsive regulators that have evolved with flowering plants

Plant invertase inhibitors are small proteins that have been identified in a variety of plant species, such as tobacco^23,24^, *Arabidopsis*^25^, maize^26^, tomato^27^, potato^28^, and sugarcane^29^. Wan et al.^12^ reported that INHs evolved in vascular plants; our BLASTP results support this, as we did not identify any INHs from lower plant species such as algae and mosses (Fig. 1a, b). Moreover, in *Amborella trichopoda*, the common ancestor of living angiosperms^30^, no *INH* gene was predicted, which supports the thesis that INHs evolved in flowering plants. Although the number of *INH* genes varies among species, *INH* gene duplication appears to have followed species evolution. The number of INHs varies in different species; for example, there are six in *Fragaria vesca*, four in *Solanum tuberosum*, two in *Solanum lycopersicum*, one in *Arabidopsis halleri*, and zero in *Amborella trichopoda*, indicating their importance in modern vascular plants. Amino acid sequence alignments of INHs from different species showed that all contained four highly conserved cysteine (Cys) residues, a hallmark of plant INHs^16^. The nucleic acid sequences, however, vary among species, even within a species (Fig. 1c).

CBF is a type of AP2/ERF transcription factor; it binds to CRT/DRE cis*-*elements in the promoter region of target genes, regulating their expression. In wild-type *Arabidopsis*, 302 genes are induced by low temperature, but in *Arabidopsis* constitutively expressing CBF, only 85 genes are induced by low temperature. Moreover, among genes downstream of CBF, 8 were inhibited when CBF was overexpressed, and these 8 genes were downregulated by low temperature^31^. These results suggest that CBF plays a key role in the plant cold response. We found numerous CRT/DRE cis-elements in 5 *PpINH* promoters (Fig. 2), indicating that these *PpINH*s are likely to be targets of CBF. The expression of *PpINH1* was significantly reduced in peach fruit during cold storage (Fig. 6f), indicating that *PpINH1* is sensitive to cold stress. These data suggest that inhibition of INH gene expression may be regulated by CBF proteins.

### PpINH1 interacts with PpVIN2 and functions as an inhibitor

VINs regulate cell growth^32^, participate in fruit sucrose metabolism^33,34^, and act as regulators in response to stress^16^, as exemplified by the increased VIN activity in peach fruit during cold storage^11^. Regulation of VIN by endogenous protein inhibitors occurs at both the transcriptional and translational levels^9^. In tomato, Qin et al.^35^ found that S1VIF, a tomato INH located in the vacuole, mediates fruit maturation and interacts with VIN S1VI, according to Y2H assays. To date, no studies have been conducted on the post-translational regulation of inhibitor-mediated VIN activity in peach fruit or other rosaceous plants. In our study, we found that, in peach fruit, only PpINH1 interacted with PpVIN2 according to the Y2H assay. We also demonstrated this interaction in tobacco cells via BiFC (Fig. 3). In potato, Brummell et al. ^28^demonstrated that INH1 is localized in the cytoplasm and cell wall, while INH2 is present in the cytoplasm and vacuole. Using a Y2H system, Lin et al.^36^ identified 27 potential StvacINV1 targets and eight StInvInh2B targets. In particular, StvacINV1 captured the Kunitz-type protein inhibitor, and their interaction was further confirmed via BiFC assays. Ji et al.^37^ proposed that the N-terminal motif of SAI is translocated into a membrane after targeting alkaline phosphatase, since its N-terminus contains a membrane localization signal. However, it cannot be excluded that there may be differences in the location of proteins, as shown by Liu et al.^38^. Overall, a short subcellular distance allows the possibility of the interaction between INH1 and VIN2.

As peach fruit are difficult to stably transform and because transgenic fruit require 4–5 years to produce, we used the *Agrobacterium* transient transformation system to investigate the biological functions of PpINH1. Our results showed that, when *PpINH1* is overexpressed in peach, VIN activity decreases (Fig. 4e), as would be expected if PpINH1 regulates VIN at the post-translational level. Zhang et al.^39^ also used an *Agrobacterium* system to generate *Sly-INH* tomato fruit. Sly-INH is an inhibitory protein that regulates tomato CWI activity at the post-translational level. Interestingly, in our experiment, although *PpINH1* expression increased substantially during cold storage, the decrease in VIN activity was not as dramatic. This discrepancy may be due to the induction of plant defense responses by *Agrobacterium* infection^40^, which would be expected to enhance VIN activity. In our study, there was a weak correlation between VIN2 expression and its activity, which may be controlled by transcriptional regulation and post-transcriptional modification^41^. Gene expression and protein transduction are time-dependent responses in different species; an increase or decrease in protein level may result from gene expression changes^42^. Differences in VIN2 expression and activity are most likely due to the presence of the protein inhibitor INH. Moreover, protein degradation and modification also affect enzyme activity after transduction.

The interaction between PpINH1 and PpVIN2 was also shown in vitro by the use of recombinant proteins. The results showed that PpINH1 inhibition of PpVIN2 activity is pH dependent, with an optimum at a pH of 4.4, and temperature dependent, with an optimum at 35 °C (Fig. 5i–l). Horthorn et al. (2010) reported that pH affects the interaction between INH and VIN; specifically, this effect is due to the INH protein containing several acidic residues^43^. It is worth noting here that most plant vacuoles are acidic, and the interaction between PpINH1 and PpVIN2 could be regulated via pH variations within the vacuole^9^. The actual pH of peach fruit during postharvest storage ranged from 3.5 to 4.8, which provides appropriate pH conditions for PpINH1 inhibition of PpVIN2 activity.

In potato, the AI *StvacINV1*, the INH *StInvInh2B*, and the sucrose non-fermentation-associated protein kinase 1 protein form a complex that regulates the activity of AI^44^. *Arabidopsis thaliana* CWIN1 and the tobacco inhibitor CIF interact to form a similar complex that has been analyzed structurally^43^. Bioinformatic analysis indicated that PpINH1 has a four-helix-bundle fold and four strictly conserved cysteine residues (Supplementary Fig. S3a), while PpVIN2 is predicted to contain a five-bladed β-propeller structure (Supplementary Fig. S3b). As these structures are prerequisites for PpINH1 and PpVIN2 to form an interaction complex^45,46^, it is likely that PpINH1 forms a complex with PpVIN2 and reduces PpVIN2 activity.

### Trehalose treatment affects PpINH1, leading to improved cold resistance of peach fruit

In most plants, the amount of endogenous trehalose is typically low; however, it is strongly induced by environmental stresses, including extreme temperatures, salinity, and drought. Therefore, endogenous trehalose may be involved not only in plant metabolism but also in signaling pathways^47^. The biosynthesis of trehalose in eukaryotes occurs through dephosphorylation of a trehalose-6-phosphate intermediate^48^. Additionally, trehalose-6-phosphate is a signaling factor that can maintain levels of sucrose (products of photosynthesis) and especially plays an important role in the process of sugar metabolism and in sugar influx in plants. A meta-analysis showed that sucrose levels were closely related to changes in trehalose-6-phosphate concentrations^49^. The application of exogenous trehalose may induce plant signaling molecules that modulate the expression of stress-responsive genes, thereby improving plant resistance to stress^21^. Kosar et al.^21^ reported that the application of exogenous trehalose improves the cold resistance of crop plants and harvested fruits, and Ding and Wang^22^ reported that 10% trehalose treatment enhances the cold tolerance of fresh-cut pepper fruits by enhancing antioxidant activity and reducing cell ultrastructure injury. The physiological or molecular regulation of such effects has not yet been reported. In this study, application of 1% trehalose to peach fruit in cold storage resulted in significantly enhanced *PpINH1* expression, reduced VIN activity, and higher sucrose contents, ultimately alleviating CI symptoms (Fig. 6). These results suggest that the upregulation of INHs inhibits VIN activity at the post-translational level in peach fruit.

Similar to overexpression of *PpINH1* inhibiting VIN in peach fruit (Fig. 4), overexpression of *StInvInh2A* and *StInvInh2B* inhibits StvacINV1 in stored tubers, alleviating cold-induced sweetening and the resulting deterioration in quality^13^. Similarly, when RNAi technology was used to silence the expression of the soybean invertase inhibitor GmCIF1 in transgenic plants, the CWI activity increased significantly. These results suggest that VIN activity may be negatively regulated by INHs in plants^50^. Additionally, overexpression of *INH2* can reduce VIN activity, and *INH2* gene silencing increases VIN activity^51^. It is inferred that VIN post-translational regulation by INH is among the important factors affecting potato low-temperature resistance^51^. The activity of the recombinant protein of VIN (TIV-1) cloned from tomato was inhibited by the protein inhibitor SolyVIF^9^. To date, research concerning VIN regulation by INH at the post-translational level has mainly focused on the phenomenon of low-temperature saccharification in potatoes, although similar studies have been carried out on tomato fruits and tobacco plants; however, this type of research has rarely been reported in peach fruit.

**Fig. 7:**
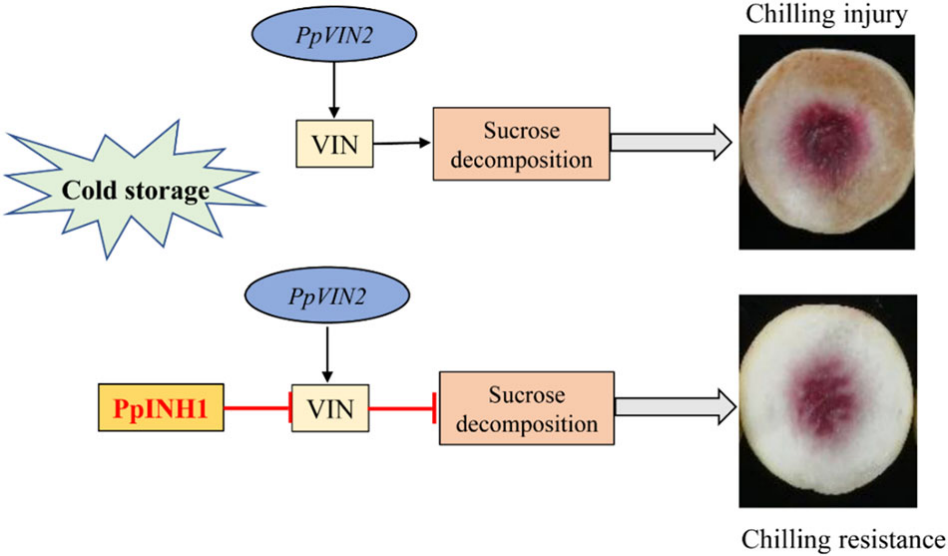
Proposed model for the inhibition of VIN activity by PpINH1. Upper panel: During cold storage, both *PpVIN2* expression and VIN activity increase. VIN breaks down sucrose, a sugar that helps prevent CI. Lower panel: PpINH1 inhibits VIN activity at the post-translational level. Trehalose treatment increases PpINH1 expression and thus indirectly increases sucrose levels, protecting against chilling injury

Based on our findings, we suggest that upregulating the expression of *PpINH1* results in decreased VIN activity, increased sucrose content, and, ultimately, enhanced chilling resistance (Fig. 7). This provides insight into the mechanism of enhancing chilling resistance by trehalose treatment.

## Conclusions

In conclusion, evolutionary analysis suggests that INHs are essential for the regulation of sucrose metabolism in vascular plants by balancing VIN activity. We demonstrated that PpINH1 interacts with PpVIN2 and inhibits VIN activity in peach fruit. Upregulation of *PpINH1* expression in trehalose-treated peach fruit resulted in decreased VIN activity, which slowed sucrose decomposition, thereby enhancing chilling resistance. Our findings provide new insight into the regulation of sucrose metabolism during chilling stress in peach fruit and have implications for the development of more effective postharvest treatment methods and for breeding fruits with cold-tolerant phenotypes.

## Materials and methods

### Plant material and treatments

Peach (*Prunus persica* L. Batsch. “Yulu”) fruits were obtained at optimum commercial maturity (~80% mature, according to growers’ recommendations) from orchards at the Fenghua Honey Peach Institute (Zhejiang, China). Fruits of uniform size, of homogeneous color, and free of disease and physical damage were selected. A total of 300 peach fruits were randomly divided into two groups. The treatment group was immersed in a 1% (m/v) trehalose solution for 10 min, and the control group was immersed in the same volume of distilled water. The fruits were then air dried and stored at 5 °C under 92–95% relative humidity. Sampling was performed after 0, 7, 14, 21, and 28 days of refrigeration. Mesocarp slices (~1 cm thick) were frozen in liquid nitrogen and stored at –80 °C. Three replicates, consisting of 10 fruits/replicate, were taken at each time point for biochemical and molecular analyses.

### Bioinformatic and evolutionary analyses

To investigate changes in INH gene expression during plant evolution, the PpINH1 protein sequence was used to query the One Thousand Plant Transcriptomes database (https://db.cngb.org/onekp/) via BLASTP. Database sequences that aligned with expectation values (*E*-values) <10^−10^ were set aside as candidates. Variations in INH gene copy number during plant evolution were investigated using the Phytozome database (https://phytozome.jgi.doe.gov/pz/portal.html). In this analysis, *PpINH1* was aligned to sequences using BLASTP (with the same significance criterion: *E*-value < 10^−10^). To analyze INH gene conservation, the nucleic acid sequences of candidate INH genes from *Amborella trichopoda*, *Prunus persica*, *Brassica rapa*, *Medicago truncatula*, and *Solanum lycopersicum* were downloaded from the Phytozome database. Finally, 19 nucleic acid sequences were aligned with ClustalW, and phylogenetic trees were constructed using the maximum likelihood method via MEGA 7.0 (https://www.megasoftware.net/), with 1000 bootstrap replicates.

Isoelectric points and molecular masses were predicted using Compute pI/Mw (https://web.expasy.org/compute_pi/). Signal peptides and locations of putative signal peptide cleavage sites were predicted using ProP 1.0 (http://www.cbs.dtu.dk/services/ProP/). The amino acid sequences of PpINH1 and PpVIN2 were compared against those of entries in the Protein Data Bank (http://www.rcsb.org/) using BLASTP. The crystal structure of a cell wall invertase inhibitor from tobacco (PDB-ID 1RJ1)^45^ with 40% sequence identity was used as a model to predict the three-dimensional structure of PpINH1. The predicted three-dimensional structure of PpVIN2 is based on the crystal structure of a 6-SST/6-SFT protein from *Pachysandra terminalis* (PDB-ID 3UGF)^52^, with 67% sequence identity. The resulting PDB file was analyzed using PyMOL (http://www.pymol.org).

### Yeast two-hybrid (Y2H) assays

Y2H assays were performed using a Matchmaker^TM^ Gold Yeast Two-Hybrid System (Clontech, USA) according to the manufacturer’s protocol. To generate a vector for Y2H analysis, full-length cDNAs of *PpVIN2* (GenBank Accession No. XM_007210252) and *PpINH*s (GenBank Accession Nos. XM_007208838, XM_007223513, XM_007209598, XM_007223389, and XM_007217302) were cloned into pGBKT7 (BD) and pGADT7 (AD) vectors, respectively. *PpVIN2* was inserted at the BamHI-PstI restriction sites of pGBKT7, yielding BD-*PpVIN2*. Each *PpINH* was then inserted at the BamHI-XhoI restriction site within pGADT7, yielding five different AD-*PpINH*s. The primers used are shown in Supplementary Table S1. The invertase and inhibitor plasmids were subsequently cotransformed into the Y2H Gold yeast strain according to the manufacturer’s instructions (Clontech, USA), and the recommended tests for autoactivation and toxicity were performed. The transformants were plated onto the SD-LT synthetic dropout nutrient media (SD/-Leu/-Trp) containing X-α-Gal and SD-LTHA (SD/-Leu/-Trp/-His/-Ade). pGADT7-T, pGBKT7-53, and pGBKT7-Lam were also cotransformed and analyzed separately as controls.

### Subcellular localization

To analyze the subcellular location of proteins in vivo, full-length copies of *PpVIN2* and *PpINH1* were cloned and inserted into pCAM35s-GFP plasmids at the KpnI-XbaI and BamHI-KpnI sites, respectively, generating *PpVIN2*-pCAM35s-GFP and *PpINH1*-pCAM35s-GFP fusion vectors, respectively. The *PpVIN2*-pCAM35s-GFP and *PpINH1*-pCAM35s-GFP constructs and the empty vector pCAM35s-GFP were transformed into *Agrobacterium* strain GV3101, after which the transformed bacteria were injected into the leaf epidermis of *Nicotiana benthamiana*. After incubation for 2 days, the inoculated leaves were examined by fluorescence microscopy using a laser scanning confocal microscope (FV10-ASW, Olympus, Japan).

### Bimolecular fluorescence complementation (BiFC) assays

For YFP fusion vector construction, the full-length *PpVIN2* coding DNA sequence (CDS) was synthesized and cloned into pSPYNE-35S at the XbaI-BamHI restriction sites. Similarly, the *PpINH1* CDS was synthesized and cloned into pSPYCE-35S at the *Xba*I-*Bam*HI sites. The resulting plasmids were *PpVIN2*-N-YFP (*PpVIN2*-YN) and *PpINH1*-C-YFP (*PpINH1*-YC). These constructs were transiently expressed in tobacco leaves as described by Glass et al.^53^. Fluorescence signals and bright field images were obtained with a confocal microscope. YFP and GFP emissions were measured at excitation wavelengths of 514 nm and 498 nm, respectively.

### *Agrobacterium*-based transient transformation

Assays for transient expression in peach fruit were conducted following protocols described by Zhang et al.^39^ and Li et al.^54^, with minor modifications. To generate the overexpression construct, the *PpINH1* CDS was excised from the BiFC fusion vector and ligated into a pBI121 vector, generating pBI121-*PpINH1*, which was subsequently transformed into *Agrobacterium tumefaciens* GV3101. The pBI121 vector was used as a negative control.

*A. tumefaciens* transformants (1 mL of 3 OD_600_ units) were injected into mature green “Yulu” peach fruit. Peach fruit were sampled 24, 36, 48, and 72 h after inoculation. The area of infection was a radius of ~1 cm around the injection site. At each time point, the fruits were cut into 1 cm^2^ slices ~1–2 mm thick. The expression of the GUS reporter plasmid (cotransformed with *PpINH1*) was assayed using a histochemical assay (RealTimes, China). For VIN activity and *PpINH1* expression assays, peels were removed with a scalpel, and the infected tissue was cut into pieces of ~0.5 g. The pieces were subsequently flash frozen in liquid nitrogen and stored at –80 °C.

### Quantitative real-time PCR (qRT-PCR)-based analysis

RNA isolation and cDNA synthesis were performed according to the methods of He et al.^11^. The primers used for qRT-PCR are shown in Supplementary Table S1. Translation elongation factor 2 (TEF2, JQ732180.1) was chosen as a reference gene^7^. Other experimental methods related to qRT-PCR were the same as those described by He et al.^11^.

### VIN activity assay

VIN was assayed according to the methods of Schaffer et al.^55^, with modifications. Frozen samples of mesocarp (1 g) were homogenized on ice in 5 mL of extraction buffer consisting of 100 mM sodium phosphate buffer (pH 7.5), 5 mM MgCl_2_, 2.5 mM dithiothreitol, 0.1% Triton X-100 (v/v), and 2% polyvinylpyrrolidone (PVPP, m/v). The homogenates were centrifuged at 12,000 × *g* for 20 min at 4 °C. The supernatants were then dialyzed in 0.1× extraction buffer (without PVPP) to remove soluble sugars. VIN activity was assayed using the protocol described above. One unit of VIN activity was defined as the amount of enzyme required to produce 1 µmol glucose/hour, expressed as units per gram of fresh weight.

### Recombinant protein expression and purification

To analyze the function of the PpINH1 protein in vitro, the *PpINH1* CDS was cloned into pET-32a at the KpnI-HindIII sites and then expressed in *E. coli* strain BL21 (DE3) (ComWin Biotech, Beijing, China) to generate a His-fusion protein. The cells were cultured in LB media consisting of 100 μg/mL ampicillin at 15 °C for 16 h, with shaking at 200 rpm. Expression of the recombinant protein was induced with 1.0 mM IPTG when the cells reached a density of 0.6 to 0.8 (OD_600_). The cell pellet from a 300 μL culture was lysed by sonication for 10 min in 200 μL of lysis buffer (50 mM Tris, 150 mM NaCl, 5% glycerol; pH 8.0). The lysate was then centrifuged at 15,000 rpm for 10 min, the supernatant was collected, and the PpINH1 protein was purified using a Ni column.

For the preparation of recombinant PpVIN2, the *PpVIN2* CDS was cloned directly into a pPICZαA vector at the XhoI-NotI sites. *Pichia pastoris* strain X-33 was then transformed with 10 μg of the linearized construct. For small-scale evaluation of protein expression, four transformant colonies were used to inoculate buffered glycerol-complex media (BMGY). Cells were cultured to an OD_600_ of 3.0, pelleted by centrifugation, and resuspended in BMGY at 28 °C (the OD_600_ was adjusted to 1.0). Pure methanol was added to a final volumetric concentration of 1% every 24 h for 4 days. After low-speed centrifugation, the supernatant was collected and analyzed by SDS-PAGE and western blotting. The protein was purified from the culture supernatants using a Ni column.

The concentrations of the purified proteins were determined using a micro-Bradford protein assay with bull serum albumin (Thermo Fisher, USA) as a standard. For western blot analysis, purified proteins were fractionated on a 12% SDS-PAGE gel and electrotransferred to an immobilon-P polyvinylidene difluoride membrane (Millipore). Mouse anti-His mAb (GenScript, Nanjing, China) was used as the primary antibody. Proteins were also displayed on a Coomassie Blue-stained 12% SDS-PAGE gel, after which the protein purity was estimated by densitometric analysis.

### In vitro recombinant protein inhibition assay

The VIN activity of recombinant PpVIN2 was assayed as described above and expressed as units per milligram of protein. Inhibitory effects were determined at different pH levels and temperatures by measuring the VIN activity of PpVIN2 in the presence of PpINH1. PpVIN2 plus double distilled water served as a control. The inhibition rate was calculated by the following formula: inhibition rate = [(PpVIN2 + ddH_2_O) – (PpVIN2 + PpINH1)]/(PpVIN2 + ddH_2_O), where each term refers to VIN activity measured in the presence (+PpINH1) or absence (+ddH_2_O) of the inhibitor.

The mixture was incubated at 37 °C for 30 min in assay buffers at a pH of 3.2, 3.6, 4.0, 4.4, 4.8, 5.2, 5.6, and 6.0. Sucrose was then added, and the enzyme activity was measured. These reactions were repeated at 25, 30, 35, 40, 45, 50, and 55 °C.

### Determination of CI indexes

Internal browning (IB) is a characteristic symptom of CI of peach fruit. IB was visually estimated after cutting along the axial diameter of 10 fruits per replicate. The estimates were in turn used to calculate the CI index^7^. The CI severity scores ranged from 0 to 4: 0 = no IB observed, 1 = mild IB, 2 = moderate IB, 3 = moderately severe IB, and 4 = severe IB. The CI index was calculated using the formula: CI index = [(CI score) × (number of fruits with this CI score)]/(4 × total number of fruits in each treatment).

### Determination of sucrose content

The sucrose content was measured using the method of Shao et al.^56^. Briefly, 5 g of frozen peach tissue was finely ground in a mixture of 0.5 mL of solution I (5.48% (m/v) zinc acetate:glacial acetic acid (97:3)) and 0.5 mL of solution II (potassium ferrocyanide 2.65% (m/v)). The resulting homogenate was diluted with deionized water to 25 mL and then passed through a 0.22 µm membrane filter. The sucrose content in a 20 µL aliquot of the filtrate was measured using an HPLC system (model 2695, Waters, USA) equipped with an X Brige^TM^ amide column (3.5 mm, 4.6250 mm, USA) and a refractive index detector (model 2414) Waters). The mobile phase composition was acetonitrile:water (80:20 v/v), the total flow rate was 1 mL/min, and the column temperature was 35 °C.

### Statistical analysis

A completely randomized design was used in the experiments. Statistical analyses were performed using SAS (version 8.2, SAS Institute, Cary, NC, USA). The qRT-PCR results were analyzed using the 2^−ΔΔCt^ method. Figures were prepared using OriginPro 9.0 (Microcal Software Inc., Northampton, MA, USA) and GraphPad Prism 5.0 (GraphPad Software, San Diego, CA, USA). Differences between means were evaluated with Student’s *t*-test.

## Supplementary information

Supplementary information
