# Supplementary material for: PpINH1, an invertase inhibitor, interacts with vacuolar invertase PpVIN2 in regulating the chilling tolerance of peach fruit
Source: Hortic Res. 2020 Oct 1;7:168. doi: 10.1038/s41438-020-00389-8 (PMC7527553; doi:10.1038/s41438-020-00389-8)
Supplement: Supplementary file 1 — Supplementary information [file 41438_2020_389_MOESM1_ESM.docx]

**Table S1** Sequences of primers used for qRT-PCR, Y2H vector construction, and subcellular localization.

| Orientation | Use | Sequence (5'-3') |
| --- | --- | --- |
| Forward | *PpINH1*-pGADT7 vector construct preparation | GGATCCATGGATGCCAGTCTCATTGAC |
| Reverse | *PpINH1*-pGADT7 vector construct preparation | CTCGAGTCAAGGCAATATCCTGGCAAT |
| Forward | *PpINH2*-pGADT7 vector construct preparation | GGATCCATGGGTGCCAATCTCATCGAC |
| Reverse | *PpINH2*-pGADT7 vector construct preparation | CTCGAGTCAAAGCATTGTCCTCGCAAT |
| Forward | *PpINH3*-pGADT7 vector construct preparation | GGATCCGTTTCCACACCAGGAGTTTAGAG |
| Reverse | *PpINH3*-pGADT7 vector construct preparation | CTCGAGTACATCAAAGCAATGTCCTGGC |
| Forward | *PpINH4*-pGADT7 vector construct preparation | GGATCCGCCTGCAAGACGAGTGAA C |
| Reverse | *PpINH4*-pGADT7 vector construct preparation | CTCGAGTTTCATTGGCTGAAACTGCTGC |
| Forward | *PpINH5*-pGADT7 vector construct preparation | GGATCCAAGGAAGGAAAAGATGAGGCGT |
| Reverse | *PpINH5*-pGADT7 vector construct preparation | CTCGAGTGCCGAGCTTATGTTGGCTT |
| Forward | *PpVIN2*-pGBKT7 vector construct preparation | GGATCCATGGCAGACCCAAGACCTTTTCTTC |
| Reverse | *PpVIN2*-pGBKT7 vector construct preparation | CTGCAGCATGAACGAAATCGAAATCG |
| Forward | *PpINH1*qRT-PCR | ATGTCCCACAAGGCAGTCAA |
| Reverse | *PpINH1* qRT-PCR | CAGCCGCAACATCAAGAAGAG |
| Forward | *PpVIN2* qRT-PCR | ACAAGGTCTTCCGTGGCAAA |
| Reverse | *PpVIN2* qRT-PCR | AGCAGCCCCATAAATTGCCT |
| Forward | qRT-PCR reference gene (TEF2) | TGAAGGAGAGGGAAGGTGAAAG |
| Reverse | qRT-PCR reference gene (TEF2) | GGTGTGACGATGAAGAGTGATG |
| Forward | *PpINH1*-GFP vector construct preparation | GGGGACGAGCTCGGTACCATGGATGCCAGTCTCATTGACAA |
| Reverse | *PpINH1*-GFP vector construct preparation | GTCGACTCTAGAGGATCCAGGCAATGTCCTGGCAATTG |
| Forward | *PpVIN2*-GFP vector construct preparation | GGGGACGAGCTCGGTACCATGGCAGACCCAAGACCTTTTC |
| Reverse | *PpVIN2*-GFP vector construct preparation | CATGGTGTCGACTCTAGATCTAACAAAAAATAAAGTTAAAA  AAATGAAGAACGGTG |


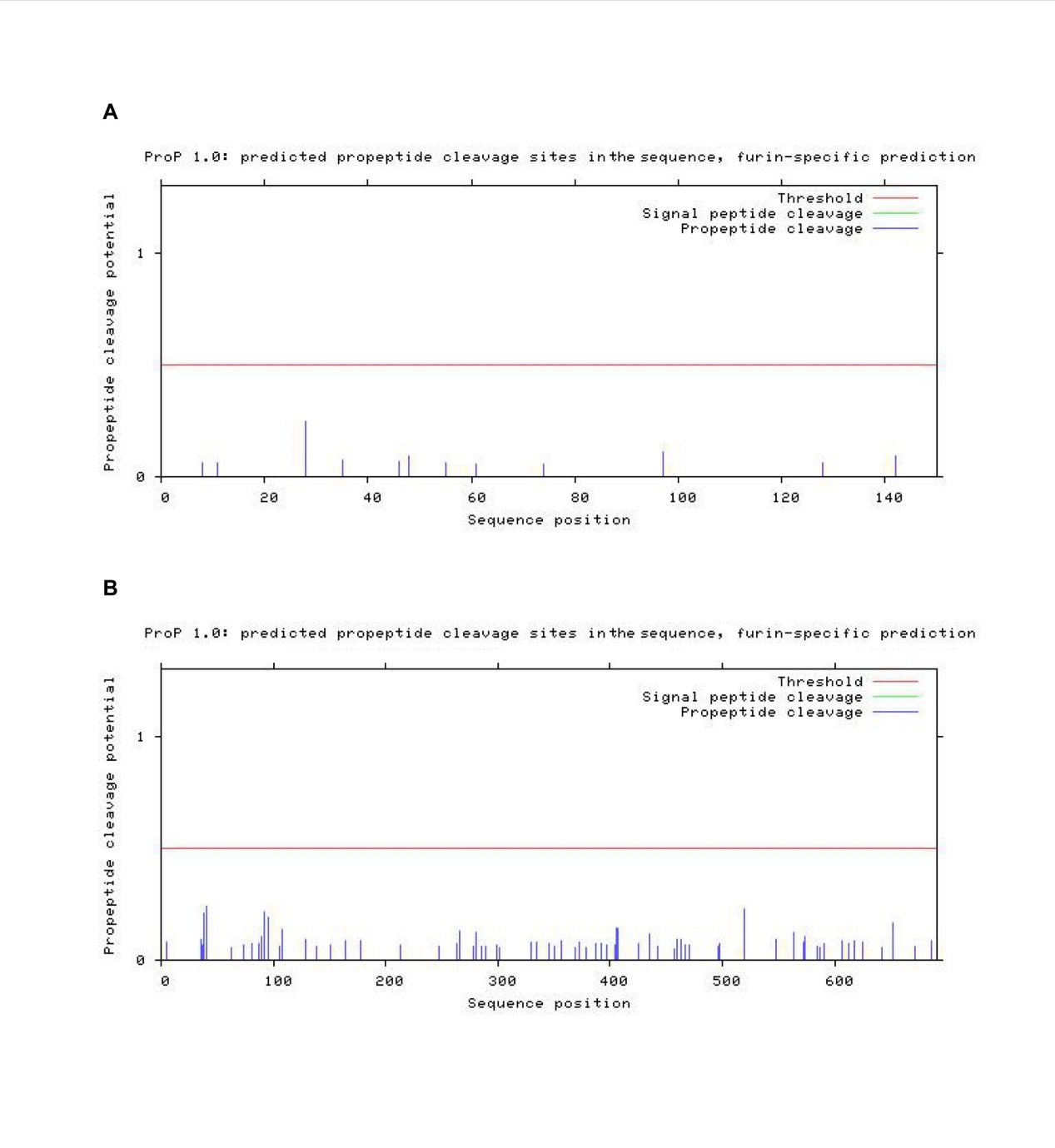
**Fig. S1** Predicted signal peptides. (**a**) PpINH1 and (**b**) PpVIN2.


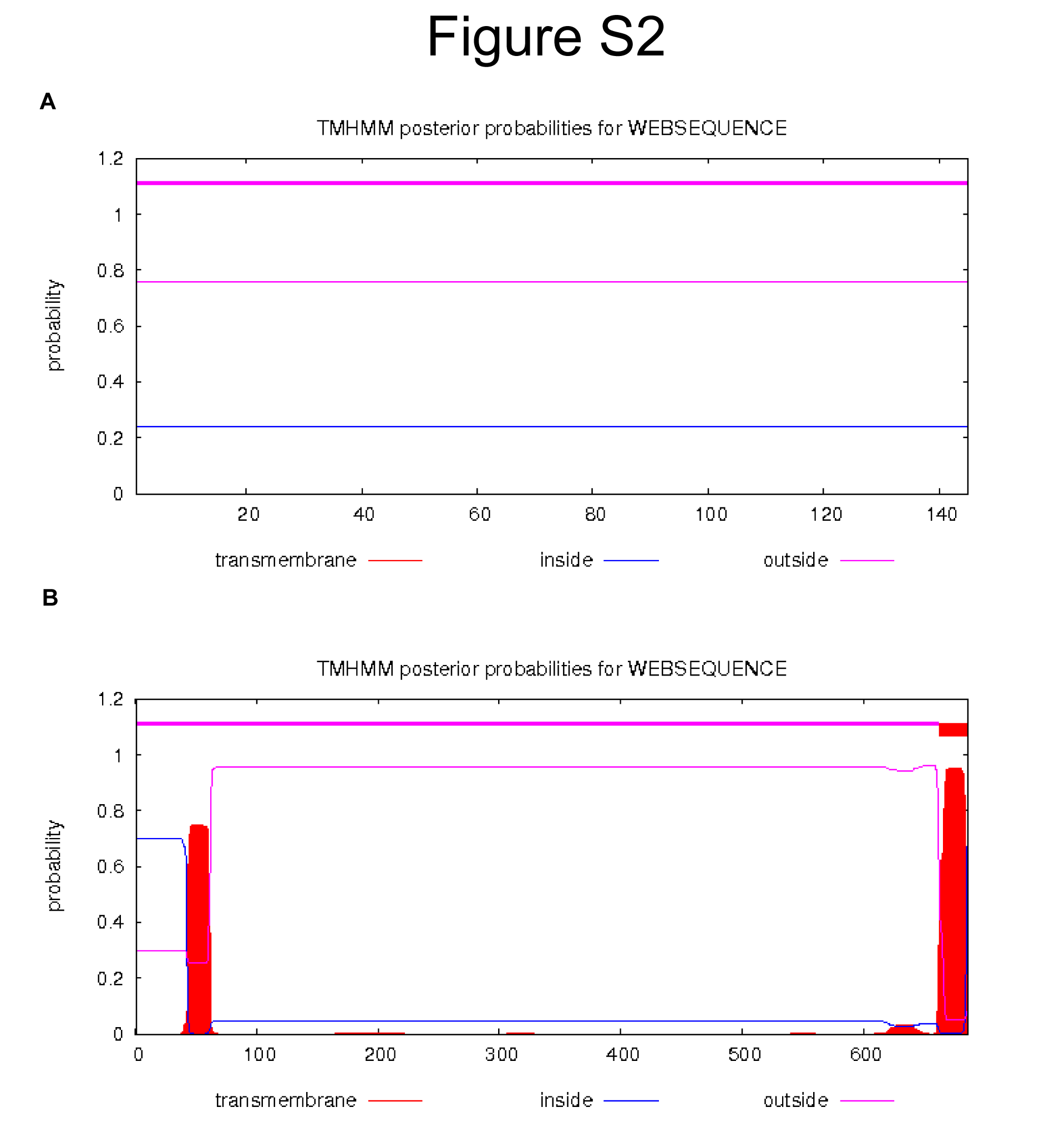


**Fig. S2** Predicted transmembrane domains in PpINH1 and PpVIN2.

(**a**) PpINH1 has no transmembrane domain. (**b**) PpVIN2 contains a single subterminal transmembrane segment located between amino acid residues 663 and 685.


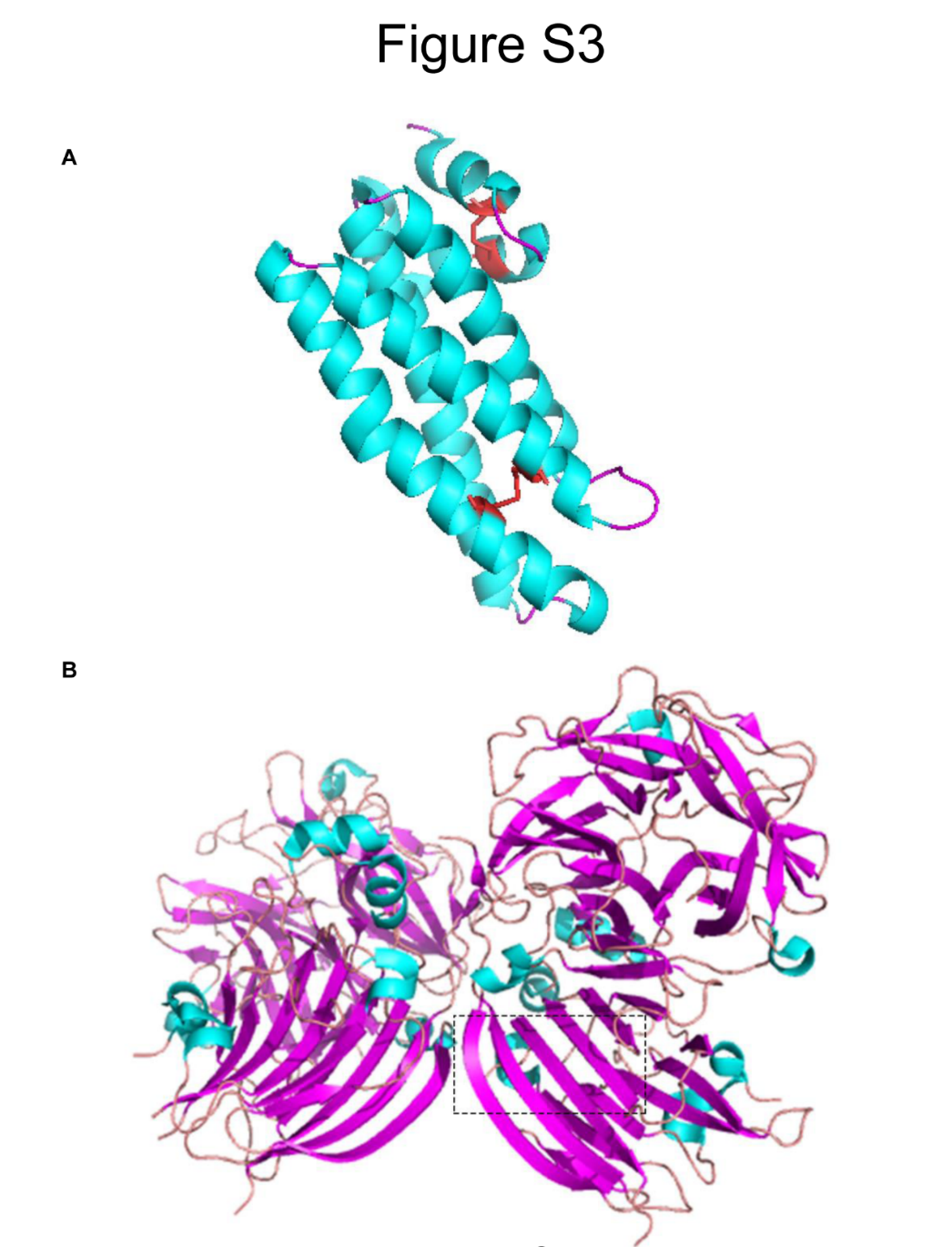


**Fig. S3** Predicted three-dimensional structure for (**a**) PpINH1 and (**b**) PpVIN2.

The blue color indicates α-helices; the purple color indicates β-folds; the five-bladed beta-propeller structure is enclosed in the dotted rectangle; and the red color indicates two disulfide bonds formed by four cysteine residues.
